# Association of cardiometabolic multimorbidity and high-risk lifestyle behaviours with subjective cognitive decline: baseline findings from the China ageing and health survey

**DOI:** 10.7189/jogh.15.04221

**Published:** 2025-11-21

**Authors:** Hongfei Zhu, Xuelan Zhao, Yurong Jing, Pengfei Wang, Zishuo Huang, Jiaoqi Ren, Houguang Zhou, Ying Wang

**Affiliations:** 1 School of Public Health, National Health Commission Key Laboratory of Health Technology Assessment and Key Laboratory of Public Health Safety of the Ministry of Education, Fudan University, Shanghai, China; 2 Department of Geriatrics, National Clinical Research Centre for Aging and Medicine, Huashan Hospital, Fudan University, Shanghai, China; 3 Department of Health Sciences, University of York, York, UK

## Abstract

**Background:**

Previous studies have reported associations between subjective cognitive decline (SCD) and both cardiometabolic multimorbidity (CMM, the co-occurrence of ≥2 cardiometabolic diseases (CMDs), including coronary heart disease, stroke, and diabetes) and lifestyle factors (LFs). While urban-rural disparities in health care access and risk factor distribution are well known, variations in these associations and the interaction between LFs and CMM among individuals with SCD in non-high-income countries remain unclear. This study aimed to investigate the association of CMM and LFs with SCD in older adults living in rural or urban areas in China.

**Methods:**

This population-based study included 41 859 older adults (median age 72.0 years; 52.48% female; 38.95% rural) from 31 provincial regions in China. Subjective cognitive decline was assessed using the Eight-Item Informant Interview to Differentiate Aging and Dementia. High-risk LFs included tobacco smoking, alcohol drinking, unhealthy diet, low physical activity, and unhealthy body shape. Cardiometabolic diseases were assessed by self-reported physician diagnoses. Lifestyle factors were collected via interviewer-administered questionnaires. Logistic regression, relative excess risk due to interaction and attributable proportion were used to assess associations and additive interactions.

**Results:**

Cardiometabolic multimorbidity (odds ratio (OR) = 2.36; 95% confidence intervals (CI) = 2.10, 2.66) and the number of CMDs (OR = 1.49; 95% CI = 1.43, 1.56) were significantly associated with an increased likelihood of SCD. Gradients in the associations between the number of high-risk LFs and SCD were observed (*P* < 0.05), except for five high-risk LFs. These associations were stronger in rural than in urban residents (*P* for interaction <0.05). Significant additive interaction was found between high-risk LFs and CMM (relative excess risk due to interaction = 1.63, 95% CI = 0.67, 2.59; attributable proportion = 0.54, 95% CI = 0.22, 0.86) for SCD.

**Conclusions:**

The coexistence of CMM and high-risk LFs exhibited an additive association with SCD. These findings highlight the need for integrated management of modifiable CMDs and lifestyle risk factors, and may inform prioritisation of rural populations.

With nearly 55.2 million people diagnosed and no cure available, dementia has become a major public health priority amid global aging, reaching epidemic proportions and incurring higher costs than most diseases [1–4]. Subjective cognitive decline (SCD) – defined as self-reported cognitive concerns without objective impairment – is one of the earliest symptomatic stages of dementia and presents a critical window for prevention [5,6]. Research shows that older adults with SCD, in the absence of objective neuropsychological dysfunction, are at increased risk of mild cognitive impairment (MCI) and eventual dementia [3,5–7]. A meta-analysis of 37 cohorts involving 70 022 older adults aged ≥60 years from North America, Europe, Australia and Asia, with 28 studies recruiting participants from the community and nine from specialist memory clinics, found an average SCD prevalence of 44% [8]. Another meta-analysis of 28 population-based cohorts indicated that individuals with SCD exhibited a conversion rate of 26.6% to MCI, and 14.1% to dementia over a four-year follow-up [9].

Cardiometabolic diseases (CMDs), including heart disease, stroke, and diabetes are linked to dementia risk [10–12]. This association may stem from shared cerebrovascular risk factors, interactions among metabolic disorders, and effects of proinflammatory state [10,13–17]. Cardiometabolic multimorbidity (CMM), defined as the coexistence of two or three CMDs, has been associated with adverse outcomes, including reduced life expectancy [18], dementia, and cognitive decline [19–23]. Most studies to date have focused on high-income countries such as those in North America and Europe. However, Liu et al. found that CMM was associated with dementia, Alzheimer disease (AD), and vascular dementia among older adults in rural China [24]. Dementia, MCI, and AD are more prevalent in rural China, likely due to disparities in health care access, education, employment services, and social networks [25–27]. Given these global rural-urban health disparities in dementia risk [28], it remains unclear whether the association of CMM with SCD differs by residence in non-high-income countries.

Furthermore, definitions of CMM regarding included CMDs vary across studies, complicating comparisons. A meta-analysis reported that association between CMM and dementia risk varies by CMM definition, with hazard ratios ranging from 2.49 (co-occurring heart disease and stroke) to 3.77 (combination of diabetes, heart disease, and stroke) [29]. While most studies included ischemic heart disease, stroke, and diabetes [11,18,30–32], the China Kadoorie Biobank study (n = 512 723; age 30–79) broadened the definition using hierarchical cluster analysis to include coronary heart disease (CHD), stroke, diabetes, and hypertension [33]. In 2023, China’s National Centre for Cardiovascular Disease reported 11.39 million CHD cases, with mortality rates of 135.08 and 148.19 per 100 000 for urban and rural residents, respectively in 2021 [34]. Given Asia’s higher CHD prevalence compared to other continents [35,36], inconsistent definitions may bias estimates of the CMM-SCD association.

Previous studies have shown that lifestyle factors (LFs), including physical activity, smoking, alcohol consumption, diet and body mass index (BMI), positively influence transitions from healthy status to CMD, CMM, and death [31,37]. Additionally, a healthy lifestyle is associated with better cognitive function and lower dementia risk [38–40]. However, few studies [21,41] have assessed the effect of LFs and CMM on cognitive function, and their findings were inconsistent, possibly due to the heterogeneity of the study populations and CMM definition. To our knowledge, no large-scale study has examined whether high-risk LFs modify the association between CMM and SCD in a Chinese population.

In this cross-sectional analysis of baseline data from a nationally representative cohort, we sought to investigate whether:

1. CMM and high-risk LFs are independently associated with SCD, with possible urban-rural differences

2. their interaction further increases the likelihood of SCD.

## METHODS

### Study participants

The study used baseline data from the China Ageing and Health Survey (CAHS), a nationwide cohort study of the Chinese older adult population. The CAHS study engaged adults aged ≥65 years at baseline in 2024 from 22 provinces, five autonomous regions, and four municipalities directly under the central government. Participants were selected using multistage, stratified probability-proportionate-to-size sampling to ensure a representative sample. The data from the baseline survey comprised 41 859 older adults, all of whom were included in this study. Data regarding SCD, CMM, LFs and other relevant variables were collected simultaneously using a single structured, interviewer-administered electronic questionnaire. The study adhered to the Declaration of Helsinki and was approved by the Institutional Review Board of Huashan Hospital, Fudan University (#2022-057). Written informed consent was obtained from all participants or their guardians prior to enrolment.

### Definition of cardiometabolic multimorbidity

Based on prior studies [33,42] and previous hierarchical cluster analysis of CAHS data, CMM was defined as the coexistence of two or three CMDs: CHD, stroke, and diabetes. Patients with CHD included those with myocardial infarction, angina pectoris, and other clinically diagnosed coronary artery diseases. Diseases were assessed by self-reported physician diagnoses.

### Measurement of subjective cognitive decline

Symptoms of SCD were assessed using the Eight-item Interview to Differentiate Aging and Dementia (AD8) [43,44], which measures self-rated changes (yes or no) in cognitive performance using eight questions covering cognition-related domains of memory, problem solving, orientation, and activities of daily living. Professionally trained interviewers administered the AD8 instrument through face-to-face interviews and assisted participants with low education or device-use issues. The AD8 scores range from 0 to 8, with higher scores indicating more severe subjective cognitive impairment. An AD8 score ≥2 indicated notable cognitive impairment, while a score of 0–1 was regarded as within the normal range [45]. The AD8 exhibits high correlation with clinical assessments, such as the Montreal Cognitive Assessment [43,44], and is widely used in large-scale studies [45–48] for its brevity.

### Assessment of lifestyle factors

According to previous studies [31,42,49,50], five LFs were considered, including tobacco smoking, alcohol drinking, unhealthy dietary habits, low physical activity and unhealthy body shape. High-risk LFs were defined based on both data availability and prior evidence linking them to cognitive function and CMDs in the Chinese population [51–54]. In the baseline questionnaires, ever-smokers (including former and current smokers) were asked how often they smoked and the amount of tobacco smoked per day; for former smokers, the year since quitting was asked. Ever-smokers were assigned to high-risk group.

Questions on alcohol consumption included number of years of drinking, whether they were abstinent or not, drinking frequency, type of alcohol (white wine, red wine, beer) and amount consumed per occasion. The high-risk group included those consuming ≥30 g/d of pure alcohol or former drinkers.

Leisure time activities, household activities, work-related activities, and total physical activity were measured using the Physical Activity Scale for the Elderly [55], scored 0–502. Leisure time (<2.14, 2.14–17.2, >17.2) and household activities (<50, 50–106, >106) were classified into light, moderate, and vigorous by quartiles [56,57]. Work-related activities, defined as paid or voluntary work, were categorised as ‘no’ or ‘yes’ since only 2.47% of participants reported engagement in this type of activity and received a score >0. Participants met WHO physical activity recommendation if they spent 2.5 hours or more per week in moderate-intensity activity, 1.25 hours or more in vigorous-intensity activity, or an equivalent combination of moderate- and vigorous-intensity activity; otherwise, they were regarded as having low physical activity levels [58,59].

The Mini-Nutritional Assessment scale was used to assess nutritional status in CAHS study, with scores ranging 0–30 [60]. Based on the responses to the questions ‘Selected consumption markers for protein intake’ and ‘Consumes two or more servings of fruit or vegetables per day,’ according to related dietary guidelines [61,62], participants were considered to have unhealthy dietary habits if they met any of the following criteria:

1. non-daily eating of at least one serving of dairy products, meat, fish, or poultry, as well as non-weekly eating of two or more servings of legumes or eggs

2. non-daily eating of two or more servings of vegetables or fruit.

Although the Mini-Nutritional Assessment-derived dietary items allow the assessment of basic dietary adequacy, they do not encompass the full spectrum of factors necessary for a comprehensive diet quality assessment.

Participation’s BMI was automatically derived by the electronic questionnaire system from self-reported height and weight. Participants with a BMI<18.5 or ≥28.0 kg/m^2^ were considered to have an unhealthy body shape, with emphasis on avoiding extreme weight and abdominal obesity [63].

### Statistical methods

For categorical variables, participant characteristics are expressed as frequencies (%); for continuous variables with skewed distribution, participant characteristics are expressed as median (interquartile range). The characteristics were compared by subjective cognitive status (AD8 ≥ 2 *vs*. AD8 < 2) using the χ^2^ test for categorical variables, and the Mann-Whitney test for continuous variables with skewed distribution. For categorical variables with expected frequencies <5, Fisher exact test was used.

For CMM, number of CMDs (0–3), presence of CMM (yes or no), and individual CMDs (CHD, stroke, and diabetes) were analysed. Each of the five high-risk LFs was treated as a binary variable (tobacco smoking, alcohol drinking, unhealthy dietary habits, low physical activity, and unhealthy body shape; presence or absence) based on their respective definitions. The number of high-risk LFs per participant was calculated as a continuous variable (ranging 0–5). Additionally, participants were grouped by presence (≥1) or absence (0) of any high-risk LFs to examine potential additive interactions. Logistic regression model was used to estimate the odds ratios (ORs) and 95% confidence intervals (CIs) for the associations of CMM and LFs with SCD. The models were stratified by rural-urban location, and adjusted for the same set of covariates in both strata: age, sex, education, marital status, smoking, drinking, physical activity, hypertension, and socioeconomic deprivation. According to a previous study [64], socioeconomic deprivation was categorised as ‘high’ when participants simultaneously met the three criteria: low education level (<6 years), manual labour occupation, and average family income per capita (≤3 000 Chinese Yuan per month).

We also examined potential additive interaction between CMM and high-risk LFs on SCD. The relative excess risk due to interaction (RERI) and the attributable proportion of interaction (AP) were used to examine additive interaction, 95% CIs were derived using the delta method, and additive interactions were considered significant if both CIs excluded zero [65].

Sensitivity analysis was conducted, with further stratification by sex, age, education level, marital status, and hypertension to test for interactions using likelihood ratio tests to compare models with and without a cross-product term. All analyses were performed using *R*, version 4.3.3 (*R* Foundation for Statistical Computing, Vienna, Austria). *P* < 0.05 indicated statistical significance.

## RESULTS

### Descriptive analysis

**Figure 1:**
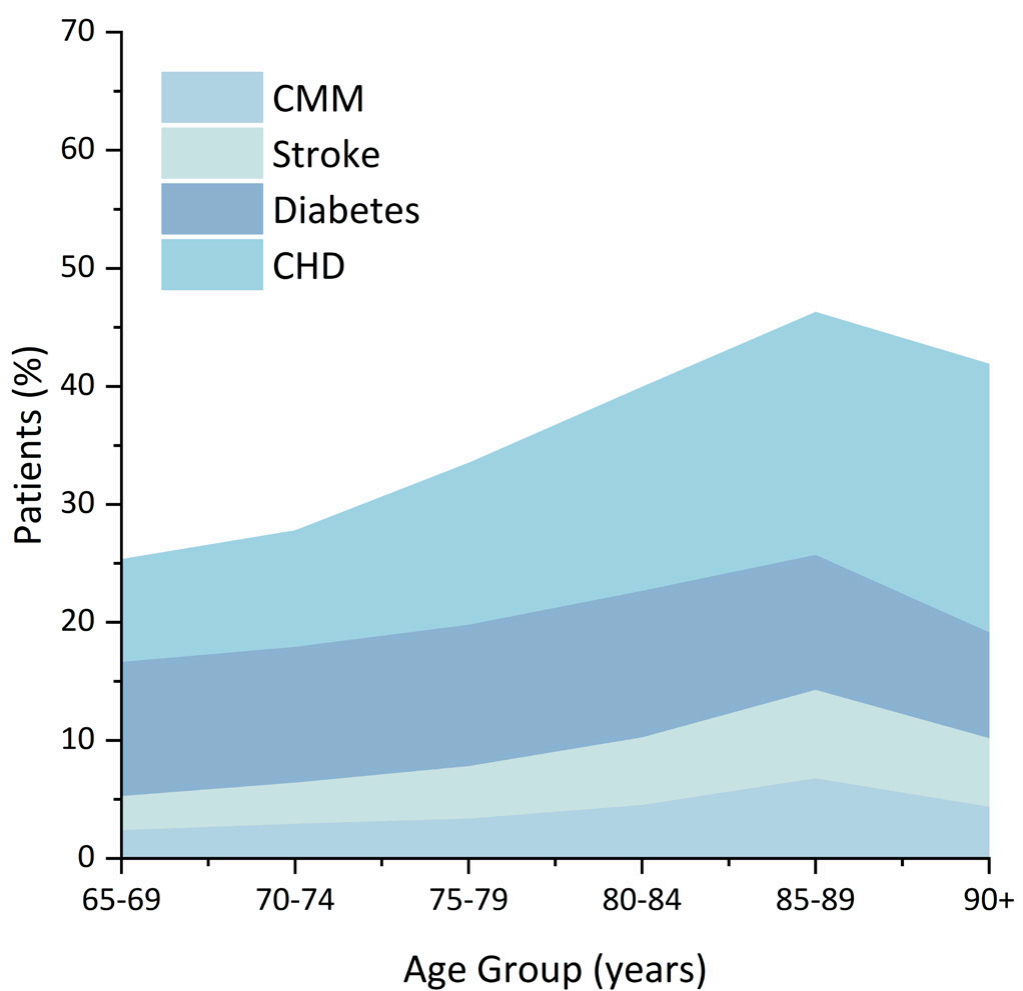
Percentage of cardiometabolic disease according to age groups. CHD – coronary heart disease, CMM – cardiometabolic multimorbidity.

Among 41 859 participants, the median age was 72 years (interquartile range = 9), 21 966 (52.48%) were female, 9418 (22.50%) were illiterate, and 16 306 (38.95%) resided in rural areas (Table S1 in the **Online Supplementary Document**). The prevalence of AD8 ≥ 2 was 29.02%. The overall prevalence of CMM in the total sample and AD8 abnormality group was 3.26 and 5.71%, respectively. The proportion of patients with CMM increases with age (**Figure 1**). Compared with AD8 normality participants, those with SCD were more likely to be female, less educated and unmarried (*P* < 0.001). Among all participants, 10 161 (24.28%) were diagnosed of at least one CMD, and 1363 (3.26%) had CMM. Overall, most participants had one or more high-risk lifestyles, and participants with SCD were less likely to meet the WHO recommendation for healthy physical activity (*P* < 0.001).

### Associations of CMDs and CMM with SCD

After controlling for potential confounding factors, the number of CMDs (OR = 1.49; 95% CI = 1.43, 1.56), presence of CMM (OR = 2.36; 95% CI = 2.10, 2.66), and three individual CMDs, including CHD (OR = 1.56; 95% CI = 1.46, 1.67), diabetes (OR = 1.27; 95% CI = 1.19, 1.36), and stroke (OR = 2.41; 95% CI = 2.17, 2.69), were significantly associated with increased likelihood of SCD (**Table 1**).

**Table 1:** Associations of cardiometabolic multimorbidity or individual cardiometabolic diseases with subjective cognitive decline

Variable	Total sample, OR (95% CI)*	Area, OR (95% CI)*		
		**Rural area**	**Urban area**	***P* for interaction**
No. of CMDs (Ref: 0)				
*1*	1.41 (1.33, 1.49)†	1.50 (1.37, 1.64)†	1.36 (1.27, 1.46)†	<0.001
*2*	2.52 (2.22, 2.86)†	4.67 (3.73, 5.83)†	1.76 (1.50, 2.07)†	
*3*	3.78 (2.46, 5.82)†	1.85 (0.67, 5.08)	4.52 (2.79, 7.33)†	
Ordinal scale	1.49 (1.43, 1.56)†	1.71 (1.59, 1.84)†	1.38 (1.30, 1.45)†	<0.001
Presence of CMM (Ref: No)	2.36 (2.10, 2.66)†	4.05 (3.26, 5.02)†	1.76 (1.52, 2.05)†	<0.001
CHD (Ref: No)	1.56 (1.46, 1.67)†	1.79 (1.60, 2.00)†	1.44 (1.32, 1.57)†	<0.001
Diabetes (Ref: No)	1.27 (1.19, 1.36)†	1.36 (1.21, 1.53)†	1.24 (1.14, 1.35)†	<0.001
Stroke (Ref: No)	2.41 (2.17, 2.69)†	2.94 (2.49, 3.46)†	2.00 (1.73, 2.31)†	<0.001

CHD – coronary heart disease, CI – confidence interval, CMDs – cardiometabolic diseases, CMM – cardiometabolic multimorbidity, OR – odds ratio

*Multivariable models were stratified by area, and adjusted for age, sex, education, marital status, hypertension and socioeconomic deprivation.

†Values are statistically significant (*P* < 0.05).

Further analysis stratified by area indicated that the associations of CMDs number (OR = 1.71; 95% CI = 1.59, 1.84), CMM (OR = 4.05; 95% CI = 3.26, 5.02), CHD (OR = 1.79; 95% CI = 1.60, 2.00), diabetes (OR = 1.36; 95% CI = 1.21, 1.53), and stroke (OR = 2.94; 95% CI = 2.49, 3.46) with increased likelihood of SCD were stronger in participants living in rural areas.

### Associations of LFs with SCD

When LFs were combined and potential confounders were controlled for, gradient in the associations between the number of high-risk LFs and increased likelihood of SCD was observed, except for the coexistence of five high-risk LFs. The adjusted OR per one-factor increase was 1.07 (95% CI = 1.03, 1.11). For individual high-risk LFs, we found that unhealthy body shape (OR = 1.08; 95% CI = 1.01, 1.16) and low physical activity (OR = 1.23; 95% CI = 1.09, 1.38) were significantly associated with SCD (**Table 2**).

**Table 2:** Associations of high-risk lifestyle factors with subjective cognitive decline

Variable	Total sample, OR (95% CI)*			Area, OR (95% CI)*			
				**Rural**		**Urban**	***P* for interaction**
No. of high-risk lifestyle factors (Ref: 0)							
*1*		1.27 (1.09, 1.48)†	1.33 (1.03, 1.72)†		1.22 (1.01, 1.48)†		<0.001
*2*		1.32 (1.13, 1.55)†	1.33 (1.02, 1.72)†		1.31 (1.08, 1.60)†		
*3*		1.38 (1.15, 1.65)†	1.33 (0.99, 1.78)		1.41 (1.12, 1.77)†		
*4*		1.87 (1.35, 2.48)†	2.00 (1.22, 3.29)†		1.81 (1.17, 2.80)†		
*5*		1.89 (0.44, 8.19)	0.92 (0.09, 9.21)		3.20 (0.43, 23.83)		
Ordinal scale		1.07 (1.03, 1.11)†	1.04 (0.98, 1.10)		1.10 (1.04, 1.15)†		<0.001
Tobacco smoking (ref: no)		1.08 (0.99, 1.17)	0.98 (0.87, 1.11)		1.16 (1.04, 1.29)†		<0.001
Alcohol drinking (ref: no)		1.05 (0.95, 1.16)	1.09 (0.94, 1.26)		1.00 (0.88, 1.15)		<0.001
Unhealthy dietary habits (ref: no)		0.89 (0.78, 1.02)	0.84 (0.68, 1.04)		0.95 (0.79, 1.13)		<0.001
Unhealthy body shape (ref: no)		1.08 (1.01, 1.16)†	1.06 (0.95, 1.19)		1.10 (1.01, 1.21)†		<0.001
Low physical activity (ref: no)		1.23 (1.09, 1.38)†	1.24 (1.01, 1.52)†		1.22 (1.04, 1.42)†		<0.001

CI – confidence interval, OR – odds ratio

*Multivariable models were stratified by area, and adjusted for age, sex, education, marital status, hypertension and socioeconomic deprivation. For analyses of individual high-risk lifestyle factors, five lifestyle factors were mutually adjusted.

†Values are statistically significant (*P* < 0.05).

There was also a gradient in the association between the number of high-risk LFs from one (OR = 1.22; 95% CI = 1.01, 1.48), two (OR = 1.31; 95% CI = 1.08, 1.60), three (OR = 1.41; 95% CI = 1.12, 1.77) to four (OR = 1.81; 95% CI = 1.17, 2.80) and increased likelihood of SCD in our stratified analysis among people residing in urban areas, the adjusted OR per one-factor increase was 1.10 (95% CI = 1.04, 1.15). Among urban residents, we found that tobacco smoking (OR = 1.16; 95% CI = 1.04, 1.29), unhealthy body shape (OR = 1.10; 95% CI = 1.01, 1.21), and low physical activity (OR = 1.22; 95% CI = 1.04, 1.42) were significantly associated with SCD. For rural residents, associations between having one (OR = 1.33; 95% CI = 1.03, 1.72), two (OR = 1.33; 95% CI = 1.02, 1.72), four (OR = 2.00; 95% CI = 1.22, 3.29) high-risk LFs and SCD were observed. Except for low physical activity (OR = 1.24; 95% CI = 1.01, 1.52), no association was found between other individual high-risk LFs with SCD.

We also observed that compared with light level of leisure time activities, moderate level (OR = 1.18; 95% CI = 1.12, 1.25) was associated with an increased likelihood of SCD, while a vigorous level (OR = 0.78; 95% CI = 0.73, 0.83) was associated with a lower risk of SCD. For household activities, both moderate (OR = 0.82; 95% CI = 0.77, 0.88) and vigorous level (OR = 0.83; 95% CI = 0.79, 0.88) were negatively associated with SCD. No significant association was observed between work-related activities and SCD (Table S2 in the **Online Supplementary Document**).

### Additional analysis and sensitivity analyses

**Table 3** shows the results of the interaction analysis between high-risk LFs and CMM for SCD. Compared to patients without both CMM and high-risk LFs, patients with CMM with high-risk LF(s) had significantly higher risk of SCD (OR = 3.00; 95% CI = 2.47, 3.63). A significant additive interaction was found between having high-risk LF(s) and CMM (RERI = 1.63; 95% CI = 0.67, 2.59; AP = 0.54; 95% CI = 0.22, 0.86) for SCD. Further analysis stratified by area showed similar results for participants living in rural areas. While no significant additive interaction between high-risk LFs and CMM was found for SCD among urban residents, tobacco smoking exhibited a significant additive interaction with CMM for SCD when individual LFs were considered (Table S3 in the **Online Supplementary Document**).

**Table 3:** The additive interaction analysis between cardiometabolic multimorbidity and high-risk lifestyle factors for subjective cognitive decline

Variables	CMM absent	CMM present
	OR (95% CI)*	OR (95% CI)*
**Total sample**		
High-risk LF(s) absent	1.00 (reference)	1.11 (0.39, 3.19)
High-risk LF(s) present	1.26 (1.08, 1.46)†	3.00 (2.47, 3.63)†
RERI (95% CI)	1.63 (0.67, 2.59)†	
AP (95% CI)	0.54 (0.22, 0.86)†	
**Rural area**		
High-risk LF(s) absent	1.00 (reference)	0.99 (0.10, 9.98)
High-risk LF(s) present	1.29 (1.00, 1.67)†	5.31 (3.81, 7.40)†
RERI (95% CI)	4.03 (1.70, 6.35)†	
AP (95% CI)	0.75 (0.32, 0.86)†	
**Urban area**		
High-risk LF(s) absent	1.00 (reference)	1.21 (0.38, 3.97)
High-risk LF(s) present	1.23 (1.02, 1.49)†	2.19 (1.72, 2.78)†
RERI (95% CI)	0.74 (−0.25, 1.72)	
AP (95% CI)	0.34 (−0.12, 0.79)	

AP – attributable proportion, CI – confidence interval, CMM – cardiometabolic multimorbidity, LF(s) – lifestyle factor(s), OR – odds ratio, RERI – relative excess risk due to interaction

*Multivariable models were stratified by area, and adjusted for age, sex, education, marital status, hypertension and socioeconomic deprivation.

†Values shown in bold are statistically significant (*P* < 0.05).

The results were not substantially altered in the sensitivity analyses. Although several statistically significant interactions were found in the stratified analyses, most of them seemed clinically meaningless (Table S4–5 in the **Online Supplementary Document**).

## DISCUSSION

This cross-sectional study, based on baseline data from a population-based cohort of rural and urban older people from 31 provincial administrative regions in China, found that both the number of CMDs and the presence of CMM were strongly associated with an increased likelihood of SCD. In addition to the coexistence of five high-risk LFs, an increased number of high-risk LFs was also significantly associated with an increased likelihood of SCD. Overall, the observed associations differed between rural and urban residents, with the likelihood of SCD for rural residents being more strongly associated with CMMs and LFs. The hypothesis of a significant additive interaction of CMM and high-risk LFs for SCD was supported. Approximately, 54% of the increased likelihood of SCD cases among participants with both high-risk LF(s) and CMM could be attributed to the interaction between these two factors, and in rural areas the interaction effect accounted for 75% of the overall effect. These additive interactions suggested a much larger effect than estimates from previous studies: based on the National Health and Nutritional Examination Survey data, Zhou et al. [66] reported a 50% attributable proportion (AP = 0.50; 95% CI = 0.25, 0.75) for the interaction of chronic kidney disease-depression synergy on cognitive impairment, while Jang et al. [67] analysed the Korean National Health Insurance Service-National Sample Cohort data, documented an 8% attributable interaction (AP = 0.08; 95% CI = 0.01, 0.46) between depression and cerebrovascular disease in increased dementia risk.

This study findings are consistent with previous cross-sectional and longitudinal research on the associations between LFs and cognitive function, as well as between CMM and cognitive function. An increasing number of CMDs have been found to be dose-dependently associated with cognitive function decline [20,21]. Therefore, CMM may represent a high-risk marker for SCD prevention. The association of cognitive decline with CMM may be explained by brain injuries caused by:

1. inflammatory cytokine release due to CMDs [68]

2. reduced cerebral blood flow or disruption of the blood-brain barrier due to damage from cardiac disease-related vascular pathology [69,70]

3. oxidative stress, accumulation of advanced glycosylation end products, vascular damage, and cerebrovascular micro-loading due to diabetes [21,71]

4. changes in cerebral blood flow and oxygen supply caused by stroke-related cerebrovascular lesions [69,72].

In our study, the relevance of the number of high-risk LFs and SCD was observed, which is consistent with previous studies. These findings confirms that there exists an additive interaction between CMM and high-risk LFs for SCD, similar to that reported in the Finnish Geriatric Intervention Study to Prevent Cognitive Impairment and Disability [73], it emphasises the possibilities this affords for multidomain management of modifiable CMDs and lifestyle-related risk factors. A substantial number of systematic reviews and meta-analyses [74,75] have identified that several modifiable LFs were beneficial for cognitive function in older adults, including smoking, alcohol consumption, diet and physical activity. Furthermore, the combination of healthy LFs has potentially additive effects on cognitive function [76]. Although the exact mechanisms remain unclear, and different LFs might have various underlying mechanisms, one potential pathway may be that LFs could supply cognitive reserve (CR) that mitigates the relationship between age-related brain changes or pathology related to AD and cognition [75,77]. Healthy LFs may increase CR, which may help people to be more resilient to physiological changes in relation to cognitive function [40].

For individual LFs, physical activity and body shape were significantly associated with cognitive function in this study after adjusting for possible confounds, emphasising the importance of these two factors in cognitive health maintenance. Physical activity may be related to cognition through various mechanisms, with potential explanations including its neuroprotective effects, such as neurogenesis induction, inflammation reduction and neuroplasticity enhancement [78–81]. Another possible explanation is the influence on neurotransmitter levels, as physical activity may increase the levels of brain-derived neurotrophic factor and insulin-like growth factor, which could enhance hippocampal plasticity and improve memory [82–84]. Previous research has reported that BMI may contribute to cognitive decline through multiple interrelated pathways. Dysregulation of hormones and chronic inflammation derived from adipose tissue, particularly involving the accumulation of amyloid-beta, interleukin-6 and C-reactive protein, have been linked to neurodegenerative processes [85–88]. These metabolic disturbances may be associated with structural brain changes such as white matter abnormalities, disruptions of the blood-brain barrier, and age-related metabolic imbalances of protein, carbohydrate, and lipid pathways [85,88]. In addition, for people living in urban areas, there may also be an emphasis on smoking cessation management. The results of this study did not reveal significant association between alcohol drinking and diet with SCD, which may be due to the lack of more precise information defining high-risk assessment criteria [41], such as years of abstinence from alcohol, and measures of dietary patterns that are beneficial in preventing CMDs or cognitive decline, such as the Mediterranean diet, Dietary Approaches to Stop Hypertension diet and Mediterranean-Dietary Approaches to Stop Hypertension Intervention for Neurodegenerative Delay diet [89,90]. Further studies are needed to clarify the criteria for assessing high-risk LFs, enabling the development of targeted intervention strategies and tools to identify high-risk populations more accurately.

Furthermore, in line with findings related to dementia [24,28], it emphasises the rural disparities in SCD prevention and CMM management. According to the Multimodal Interventions to Delay Dementia and Disability in Rural China project, older people from rural areas in China tend to be of low socio-economic status and have little or no formal education, and this socio-demographic group has been significantly under-represented in dementia research [91]. In terms of the social environment, there are differences between urban and rural areas in terms of basic community infrastructure, number of days when roads are impassable, outdoor exercise facilities, and average participation in social activities. These community environment factors have a greater impact on the cognitive decline of rural older people [26].

This study has certain limitations that warrant consideration. First, we used the baseline data of CAHS study; however, the cross-sectional data cannot be used to infer causal relationships. More information may be available from longitudinal follow-up. Assessing the dynamic relationships between lifestyle, CMM, and cognitive function is particularly complex because the magnitude of lifestyle changes may be difficult to quantify as beneficial or harmful, and past LFs may have influenced current CMM and cognitive function. These findings may inform future research adopting a life-course approach to investigate the cumulative effects of LFs across ageing stages. Second, the measurement of disease prevalence may be underestimated by self-reported diagnoses, and future studies may consider collaborating with health care organisations, where available, to confirm prevalence based on historical diagnostic data. In addition, the AD8 scale was used to assess SCD in this study. While the AD8 demonstrated good concordance with clinical assessments and neuropsychological tests in validation studies [43,44,92], its reliance on self-reported information precludes diagnostic application for cognitive dysfunction. Future research may benefit from incorporating complementary assessment approaches such as the 3-Word recall test to provide more objective data [93]. Third, there were some missing data when assessing socioeconomic deprivation; however, as an adjustment variable, it is unlikely to have influenced the study findings. Fourth, unlike overall physical activity, there are no established reference standards for categorising leisure time and household activities. Therefore, these domains were categorised according to the data distribution in this study, which may limit comparability with other studies. In addition, the cross-sectional design of the study should also be taken into account when interpreting the results of the interaction analyses, as it is not possible to capture time-varying dynamic effects.

## CONCLUSIONS

In conclusion, SCD was associated with CMM and an increasing number of high-risk LFs. There was an interaction effect between high-risk lifestyle and CMM on SCD. These findings support the need to explore integrated management of modifiable CMDs and lifestyle risk factors and may inform strategies to identify high-risk older adults, especially in rural settings.

## Additional material


Online Supplementary Document
