# Supplementary material for: Association of cardiometabolic multimorbidity and high-risk lifestyle behaviours with subjective cognitive decline: baseline findings from the China ageing and health survey
Source: J Glob Health. 2025 Nov 21;15:04221. doi: 10.7189/jogh.15.04221 (PMC12635789; doi:10.7189/jogh.15.04221)
Supplement: Online Supplementary Document [file jogh-15-04221-s001.pdf]

Supplement to: Zhu H, Zhao X, Jing Y, Wang P, Huang Z, Ren J, Zhou H, Wang Y. Association of cardiometabolic multimorbidity and high-risk lifestyle behaviours with subjective cognitive decline: baseline findings from the China ageing and health survey. J Glob Health. 2025;15:04221.

Table S1. Characteristics of study participants in the total sample and by subjective cognitive status

Table S2. Associations of leisure time, household and work-related activities with subjective cognitive decline

Table S3. The additive interaction analysis of low physical activity, unhealthy body shape or tobacco smoking with cardiometabolic multimorbidity for subjective cognitive decline

Table S4. Sensitivity analysis of associations of cardiometabolic multimorbidity or individual cardiometabolic diseases with subjective cognitive decline

Table S5. Sensitivity analysis of associations of high-risk lifestyle factors with subjective cognitive decline

Table S1. Characteristics of study participants in the total sample and by subjective cognitive status

Characteristics	Total sample(n=41,859)	Subjective cognitive status		
		AD8 abnormality (AD8 ≥2，n=12,147）	AD8 normality （AD8 <2，n=29,712）	*P* value
Age (y), median (IQR)	72.00 (9.00)	72.00 (10.00)	72.00 (8.00)	<0.001
Female, n (%)	21,966 (52.48%)	6,889 (56.71%)	15,077 (50.74%)	<0.001
Educational level, n (%)				
Illiterate	9,418 (22.50%)	3,667 (30.19%)	5,751 (19.36%)	<0.001
Primary school	13,057 (31.19%)	3,880 (31.94%)	9,177 (30.89%)	0.035
Middle school	10,402 (24.85%)	2,496 (20.55%)	7,906 (26.61%)	<0.001
High school	6,279 (15.00%)	1,526 (12.56%)	4,753 (16.00%)	<0.001
College and above	2,703 (6.46%)	578 (4.76%)	2,125 (7.15%)	<0.001
Married, n (%)	30,856 (73.71%)	8,183 (67.37%)	22,673 (76.31%)	<0.001
Socioeconomic deprivation, n (%) *				
Low	30,275 (79.57%)	8,790 (79.43%)	21,485 (79.63%)	<0.001
High	7,772 (20.43%)	2,276 (20.57%)	5,496 (20.37%)	<0.001
Area, n (%)				
Rural	16,306 (38.95%)	5,509 (45.35%)	10,797 (36.34%)	<0.001
Urban	25,553 (61.05%)	6,638 (54.65%)	18,915 (63.66%)	<0.001
Having high-risk lifestyle, n (%)				

Characteristics	Total sample(n=41,859)	Subjective cognitive status		*P* value
		AD8 abnormality (AD8 ≥2，n=12,147）	AD8 normality （AD8 <2，n=29,712）	
Tobacco smoking	5,306 (12.68%)	1,443 (11.88%)	3,863 (13.00%)	0.002
Alcohol drinking	3,023 (7.22%)	825 (6.79%)	2,198 (7.40%)	0.031
Unhealthy dietary habits	5,390 (12.88%)	1,521 (12.52%)	3,869 (13.02%)	0.170
Low physical activity	39,993 (95.54%)	11,748 (96.72%)	28,245 (95.06%)	<0.001
Unhealthy body shape	5,427 (12.96%)	1,584 (13.04%)	3,843 (12.93%)	0.782
No. of high-risk lifestyle factors, n (%)				
0	1,248 (2.98%)	265 (2.18%)	983 (3.31%)	<0.001
1	28,816 (68.84%)	8,561 (70.48%)	20,255 (68.17%)	<0.001
2	9,257 (22.11%)	2,610 (21.49%)	6,647 (22.37%)	0.049
3	2,285 (5.46%)	627 (5.16%)	1,658 (5.58%)	0.092
4	238 (0.57%)	265 (2.18%)	159 (0.54%)	0.176
5	9 (0.02%)	3 (0.02%)	6 (0.02%)	0.725
BMI, median (IQR)	23.44 (3.89)	23.44 (4.11)	23.44 (3.76)	0.768
MNA score, median (IQR)	25.00 (3.50)	25.00 (3.50)	25.00 (3.50)	0.540
PASE score, median (IQR)	99.11 (97.43)	86.86 (105.32)	104.82 (97.46)	<0.001
Hypertension, n (%)	16,995 (40.60%)	5,340 (43.96%)	11,655 (39.23%)	<0.001
CHD, n (%)	5,089 (12.16%)	2,029 (16.70%)	3,060 (10.29%)	<0.001
Stroke, n (%)	1,704 (4.07%)	905 (7.45%)	799 (2.69%)	<0.001

Characteristics	Total sample(n=41,859)	Subjective cognitive status AD8 abnormality (AD8 ≥2，n=12,147)	AD8 normality （AD8 <2，n=29,712）	*P* value
Diabetes, n (%)	4,843 (11.57%)	1,619 (13.33%)	3,224 (10.85%)	<0.001
No. of CMDs, n (%)				
0	31,698 (75.72%)	8,358 (68.81%)	23,340 (78.55%)	<0.001
1	8,797 (21.02%)	3,096 (25.49%)	5,701 (19.19%)	<0.001
2	1,253 (2.99%)	622 (5.12%)	631 (2.12%)	<0.001
3	111 (0.27%)	71 (0.58%)	40 (0.13%)	<0.001
CMM, n (%)	1,364 (3.26%)	693 (5.71%)	671 (2.26%)	<0.001

AD8 – Eight-item Interview to Differentiate Aging and Dementia, BMI – body mass index, CHD – coronary heart disease, CMDs – cardiometabolic diseases, CMM – cardiometabolic multimorbidity, IQR – interquartile range, MNA – Mini-Nutritional Assessment, PASE – Physical Activity Scale for the Elderly.

*There were 3,812 missing values for the socioeconomic deprivation variable, attributable to the fact that family income was not a mandatory field during data collection.

Table S2. Associations of leisure time, household and work-related activities with subjective cognitive decline

	Total sample, OR (95% CI)	Area, Odds ratio (95% CI)		
		Rural	Urban	*P* for interaction
Leisure-time activities (Ref: Light)				
Moderate	**1.18 (1.12, 1.25)**	**1.28 (1.18, 1.39)**	**1.12 (1.03, 1.21)**	0.037
Vigorous	**0.78 (0.73, 0.83)**	**0.86 (0.77, 0.94)**	**0.75 (0.69, 0.82)**	0.245
Household activities (Ref: Light)				
Moderate	**0.82 (0.77, 0.88)**	**0.89 (0.81, 0.98)**	**0.78 (0.72, 0.84)**	0.005
Vigorous	**0.83 (0.79, 0.88)**	**0.86 (0.78, 0.94)**	**0.81 (0.75, 0.88)**	0.091
Work-related activities (Ref: No)	1.01 (0.83, 1.21)	1.08 (0.81, 1.43)	0.95 (0.73, 1.21)	0.582

CI – confidence interval, OR – odds ratio.

*Multivariable models were stratified by area, and adjusted for age, sex, education, marital status, hypertension, socioeconomic deprivation, smoking, drinking, diet habits and body shape.

†Values shown in bold are statistically significant ($p < 0.05$).

Table S3. The additive interaction analysis of low physical activity, unhealthy body shape or tobacco smoking with cardiometabolic multimorbidity for subjective cognitive decline

	**Presence of CMM**	OR (95% CI)	RERI (95% CI)	AP (95% CI)
**Low physical activity**				
Total sample				
No	No	1.00	-0.09 (-0.20, 0.01)	-0.04 (-0.08, 0.00)
No	Yes	**2.49 (2.13, 2.90)**		
Yes	No	**1.20 (1.14, 1.26)**		
Yes	Yes	**2.59 (2.15 3.12)**		
Rural area				
No	No	1.00	-1.52 (-1.65, 1.39)	-0.43 (-0.47, -0.39)
No	Yes	**4.88 (3.70, 6.44)**		
Yes	No	**1.17 (1.08, 1.26)**		
Yes	Yes	**3.53 (2.51, 4.98)**		
Urban area				
No	No	1.00	**0.35 (0.19, 0.52)**	**0.16 (0.08, 0.23)**
No	Yes	**1.66 (1.36, 2.03)**		
Yes	No	**1.22 (1.13, 1.30)**		
Yes	Yes	**2.23 (1.78, 2.80)**		
**Unhealthy body shape**				
Total sample				

	**Presence of CMM**	OR (95% CI)	RERI (95% CI)	AP (95% CI)
No	No	1.00	**-0.42 (-0.60, -0.24)**	**-0.20 (-0.29, -0.11)**
No	Yes	**1.10 (1.02, 1.18)**		
Yes	No	**2.43 (2.14, 2.76)**		
Yes	Yes	**2.11 (1.50, 2.97)**		
Rural area				
No	No	1.00	**-0.80 (-1.02, -0.58)**	**-0.23 (-0.29, -0.17)**
No	Yes	1.08 (0.96, 1.20)		
Yes	No	**4.20 (3.34, 5.28)**		
Yes	Yes	**3.48 (1.83, 6.63)**		
Urban area				
No	No	1.00	-0.23 (-0.51, 0.06)	-0.14 (-0.31, 0.03)
No	Yes	**1.11 (1.01, 1.21)**		
Yes	No	**1.77 (1.51, 2.08)**		
Yes	Yes	**1.65 (1.09, 2.51)**		
**Tobacco smoking**				
Total sample				
No	No	1.00	**-0.72 (-0.92, -0.51)**	-0.39 (-0.50, -0.28)
No	Yes	**2.47 (2.17, 2.80)**		
Yes	No	**1.10 (1.00, 1.19)**		
Yes	Yes	**1.84 (1.31, 2.59)**		

	**Presence of CMM**	OR (95% CI)	RERI (95% CI)	AP (95% CI)
Rural area				
No	No	1.00	-0.08 (-0.33, 0.17)	-0.04 (-0.18, 0.09)
No	Yes	**4.88 (3.70, 6.44)**		
Yes	No	**1.17 (1.08, 1.26)**		
Yes	Yes	**3.53 (2.51, 4.98)**		
Urban area				
No	No	1.00	**-2.29 (-2.63, -1.94)**	**-1.07 (-1.23, -0.91)**
No	Yes	**1.02 (0.90, 1.15)**		
Yes	No	**4.40 (3.50, 5.54)**		
Yes	Yes	**2.14 (1.08, 4.23)**		

AP – attributable proportion, CI – confidence interval, CMM – cardiometabolic multimorbidity, OR – odds ratio, RERI – relative excess risk due to interaction.

*Multivariable models were stratified by area, and adjusted for age, sex, education, marital status, hypertension and socioeconomic deprivation.

†Values shown in bold are statistically significant ($p < 0.05$).

Table S4. Sensitivity analysis of associations of cardiometabolic multimorbidity or individual cardiometabolic diseases with subjective cognitive decline, OR (95%CI)

	No. of CMDs (Ref: 0)				Presence of CMM (Ref: No)	CHD (Ref: No)	Diabetes (Ref: No)	Stroke (Ref: No)
	1	2	3	Ordinal scale				
Total sample	**1.41 (1.33, 1.49)**	**2.52 (2.22, 2.86)**	**3.78 (2.46, 5.82)**	**1.49 (1.43, 1.56)**	**2.36 (2.10, 2.66)**	**1.56 (1.46, 1.67)**	**1.27 (1.19, 1.36)**	**2.41 (2.17, 2.69)**
Sex								
Female	**1.34 (1.24, 1.45)**	**2.67 (2.25, 3.16)**	**4.52 (2.52, 8.11)**	**1.48 (1.40, 1.57)**	**2.56 (2.18, 3.01)**	**1.56 (1.42, 1.71)**	**1.30 (1.19, 1.43)**	**2.31 (1.98, 2.70)**
Male	**1.50 (1.38, 1.63)**	**2.35 (1.95, 2.84)**	**2.97 (1.54, 5.73)**	**1.51 (1.41, 1.61)**	**2.14 (1.79, 2.56)**	**1.57 (1.41, 1.74)**	**1.24 (1.11, 1.38)**	**2.51 (2.16, 2.92)**
P for interaction	0.095			0.895	0.104	0.843	0.357	0.480
Education								
Illiterate	**1.85 (1.65, 2.07)**	**6.40 (4.86, 8.43)**	**4.9 (1.55, 15.49)**	**2.08 (1.91, 2.27)**	**5.34 (4.09, 6.97)**	**2.12 (1.84, 2.44)**	**1.80 (1.56, 2.07)**	**3.24 (2.68, 3.92)**
Primary school	**1.31 (1.18, 1.45)**	**1.91 (1.51, 2.42)**	**7.57 (3.04, 18.85)**	**1.38 (1.27, 1.49)**	**1.97 (1.58, 2.47)**	**1.46 (1.28, 1.65)**	**1.15 (1.01, 1.31)**	**2.13 (1.76, 2.59)**
Middle school	**1.35 (1.20, 1.52)**	**2.00 (1.52, 2.62)**	**2.41 (1.17, 4.97)**	**1.37 (1.25, 1.50)**	**1.87 (1.45, 2.41)**	**1.42 (1.23, 1.63)**	**1.21 (1.04, 1.40)**	**2.28 (1.78, 2.92)**

	No. of CMDs (Ref: 0)				Presence of CMM (Ref: No)	CHD (Ref: No)	Diabetes (Ref: No)	Stroke (Ref: No)
	1	2	3	Ordinal scale				
High school	**1.14 (0.98, 1.32)**	**1.48 (1.05, 2.09)**	**2.11 (0.69, 6.43)**	**1.18 (1.05, 1.33)**	**1.47 (1.06, 2.03)**	**1.33 (1.11, 1.59)**	0.97 (0.80, 1.16)	**1.74 (1.25, 2.43)**
College and above	1.25 (0.98, 1.58)	1.51 (0.92, 2.50)	9.76 (0.92, 103.96)	**1.28 (1.07, 1.52)**	1.56 (0.96, 2.52)	**1.34 (1.02, 1.76)**	1.22 (0.91, 1.63)	1.56 (0.88, 2.74)
P for interaction	＜0.001			＜0.001	＜0.001	＜0.001	＜0.001	0.003
Marital status								
Married	**1.44 (1.35, 1.54)**	**2.45 (2.12, 2.84)**	**3.29 (1.98, 5.47)**	**1.49 (1.42, 1.57)**	**2.56 (2.18, 3.01)**	**1.56 (1.42, 1.71)**	**1.30 (1.19, 1.43)**	**2.31 (1.98, 2.70)**
Unmarried	**1.31 (1.17, 1.47)**	**2.70 (2.12, 3.43)**	**5.34 (2.26, 12.65)**	**1.48 (1.36, 1.61)**	**2.42 (2.15, 2.72)**	**1.57 (1.47, 1.67)**	**1.28 (1.19, 1.37)**	**2.54 (2.29, 2.82)**
P for interaction	0.188			0.968	0.291	0.580	0.988	0.352
Hypertension								
No	**1.40 (1.29, 1.51)**	**2.67 (2.09, 3.42)**	**1.57 (0.61, 4.00)**	**1.44 (1.35, 1.54)**	**2.42 (1.91, 3.06)**	**1.15 (1.03, 1.28)**	**1.36 (1.22, 1.51)**	**2.73 (2.35, 3.18)**
Yes	**1.42 (1.31, 1.54)**	**2.48 (2.14, 2.87)**	**4.95 (2.98, 8.22)**	**1.52 (1.44, 1.61)**	**2.35 (2.04, 2.70)**	**1.93 (1.76, 2.11)**	**1.20 (1.10, 1.32)**	**2.12 (1.82, 2.47)**

	No. of CMDs (Ref: 0)				Presence of CMM (Ref: No)	CHD (Ref: No)	Diabetes (Ref: No)	Stroke (Ref: No)
	1	2	3	Ordinal scale				
P for interaction	0.805			0.240	0.825	＜0.001	0.110	0.015
Socioeconomic deprivation								
No	**1.39 (1.31, 1.48)**	**2.52 (2.18, 2.90)**	**3.69 (2.27, 6.00)**	**1.48 (1.41, 1.55)**	**2.36 (2.06, 2.70)**	**1.56 (1.45, 1.69)**	**1.27 (1.18, 1.38)**	**2.26 (2.00, 2.56)**
Yes	**1.47 (1.30, 1.66)**	**2.54 (1.94, 3.33)**	**4.17 (1.66, 10.48)**	**1.53 (1.39, 1.68)**	**2.38 (1.84, 3.08)**	**1.55 (1.33, 1.80)**	**1.26 (1.08, 1.48)**	**3.08 (2.42, 3.91)**
P for interaction	0.341			0.337	0.758	0.857	0.885	0.021

CHD – coronary heart disease, CI – confidence interval, CMDs – cardiometabolic diseases, CMM – cardiometabolic multimorbidity, OR – odds ratio.

*Multivariable models were adjusted for age, sex, education, marital status, hypertension and socioeconomic deprivation, and stratified by the above factors except age.

†Values shown in bold are statistically significant (p＜0.05).

Table S5. Sensitivity analysis of associations of high-risk lifestyle factors with subjective cognitive decline, OR (95%CI)

	No. of high-risk lifestyle factors	Tobacco smoking (Ref: No)	Alcohol drinking (Ref: No)	Unhealthy dietary habits (Ref: No)	Unhealthy body shape (Ref: No)	Low physical activity (Ref: No)
Total sample	**1.07 (1.03, 1.11)**	1.08 (0.99, 1.17)	1.05 (0.95, 1.16)	0.89 (0.78, 1.02)	**1.08 (1.01, 1.16)**	**1.23 (1.09, 1.38)**
Sex						
Female	**1.16 (1.08, 1.24)**	**1.35 (1.07, 1.71)**	**1.48 (1.13, 1.94)**	0.94 (0.85, 1.03)	**1.13 (1.03, 1.24)**	**1.10 (1.03, 1.18)**
Male	1.03 (0.99, 1.08)	1.07 (0.98, 1.16)	1.04 (0.94, 1.15)	1.02 (0.92, 1.13)	1.02 (0.92, 1.14)	**1.31 (1.21, 1.41)**
P for interaction	0.126	0.009	0.431	0.603	0.959	＜0.001
Education						
Illiterate	1.00 (0.92, 1.09)	0.86 (0.71, 1.05)	1.07 (0.86, 1.34)	0.93 (0.81, 1.06)	1.03 (0.90, 1.18)	1.03 (0.93, 1.13)
Primary school	**1.08 (1.01, 1.15)**	**1.14 (1.00, 1.30)**	1.16 (0.99, 1.37)	0.98 (0.86, 1.11)	1.04 (0.92, 1.18)	**1.33 (1.21, 1.45)**
Middle school and above	**1.11 (1.05, 1.17)**	**1.17 (1.05, 1.31)**	1.05 (0.92, 1.20)	1.0 (0.90, 1.11)	**1.16 (1.04, 1.30)**	**1.23 (1.13, 1.33)**
P for interaction	0.091	0.011	0.431	0.578	0.919	＜0.001
Marital status						
Married	**1.06 (1.01, 1.10)**	1.05 (0.97, 1.14)	1.06 (0.95, 1.17)	0.94 (0.87, 1.02)	1.08 (0.99, 1.17)	**1.18 (1.11, 1.25)**
Unmarried	**1.13 (1.04, 1.22)**	**1.27 (1.06, 1.51)**	1.19 (0.95, 1.49)	1.10 (0.95, 1.27)	1.11 (0.97, 1.27)	**1.24 (1.12, 1.38)**
P for interaction	0.785	0.001	0.225	0.691	＜0.001	＜0.001
Hypertension						

	No. of high-risk lifestyle factors	Tobacco smoking (Ref: No)	Alcohol drinking (Ref: No)	Unhealthy dietary habits (Ref: No)	Unhealthy body shape (Ref: No)	Low physical activity (Ref: No)
No	1.04 (0.98, 1.10)	1.06 (0.94, 1.19)	0.99 (0.86, 1.15)	0.96 (0.86, 1.06)	1.02 (0.91, 1.13)	**1.30 (1.20, 1.40)**
Yes	**1.09 (1.04, 1.15)**	**1.11 (1.00, 1.23)**	**1.15 (1.02, 1.30)**	0.99 (0.90, 1.08)	**1.14 (1.04, 1.25)**	**1.12 (1.05, 1.20)**
P for interaction	0.093	0.011	0.423	0.602	0.917	＜0.001
Socioeconomic deprivation						
No	**1.06 (1.01, 1.10)**	1.07 (0.98, 1.16)	1.08 (0.97, 1.20)	0.94 (0.87, 1.02)	**1.08 (1.00, 1.17)**	**1.19 (1.13, 1.26)**
Yes	**1.12 (1.03, 1.22)**	1.17 (0.99, 1.38)	1.08 (0.87, 1.33)	1.11 (0.95, 1.29)	1.11 (0.95, 1.29)	**1.20 (1.07, 1.34)**
P for interaction	0.123	0.009	0.528	0.588	0.856	＜0.001

CI – confidence interval, OR – odds ratio.

*Multivariable models were adjusted for age, sex, education, marital status, hypertension and socioeconomic deprivation, and stratified by the above factors except age.

†Values shown in bold are statistically significant (p＜0.05).

‡There was insufficient data to include number of high-risk lifestyle factors as a categorical variable for sensitivity analysis.
